# mTOR-Dependent Stimulation of *IL20RA* Orchestrates Immune Cell Trafficking through Lymphatic Endothelium in Patients with Crohn’s Disease

**DOI:** 10.3390/cells8080924

**Published:** 2019-08-18

**Authors:** Federica Ungaro, Valentina Garlatti, Luca Massimino, Antonino Spinelli, Michele Carvello, Matteo Sacchi, Salvatore Spanò, Gaia Colasante, Nicholas Valassina, Stefania Vetrano, Alberto Malesci, Laurent Peyrin-Biroulet, Silvio Danese, Silvia D’Alessio

**Affiliations:** 1 IBD Center, Laboratory of Gastrointestinal Immunopathology, Humanitas Clinical and Research Center, Rozzano, 20089 Milan, Italy; 2 Department of Biomedical Sciences, Humanitas University, Pieve Emanuele, 20090 Milan, Italy; 3 Division of Neuroscience, San Raffaele Scientific Institute, 20132 Milan, Italy; 4 Colon and Rectal Surgery Unit, Humanitas Clinical and Research Center, Rozzano, 20089 Milan, Italy; 5 Department of Medical Biotechnologies and Translational Medicine, University of Milan, 200129 Milan, Italy; 6 Department of Gastroenterology, Humanitas Clinical and Research Center, Rozzano, 20089 Milan, Italy; 7 Inserm Ngere and Nancy University Hospital, Lorraine University, 54500 Vandoeuvre-lès-Nancy, France

**Keywords:** Crohn’s disease, lymphatic endothelial cells, intestinal inflammation, transcriptomics

## Abstract

Crohn’s disease (CD) is a chronic inflammatory condition that can affect different portions of the gastrointestinal tract. Lymphatic drainage was demonstrated to be dysfunctional in CD pathogenesis, ultimately causing the failure of the resolution of intestinal inflammation. To investigate the molecular mechanisms underlying these dysfunctions, we isolated human intestinal lymphatic endothelial cells (HILECs) from surgical specimens of patients undergoing resection for complicated CD (CD HILEC) and from a disease-free margin of surgical specimens of patients undergoing resection for cancer (healthy HILEC). Both cell types underwent transcriptomic profiling, and their barrier functionality was tested using a transwell-based co-culture system between HILEC and lamina propria mononuclear cells (LPMCs). Results showed CD HILEC displayed a peculiar transcriptomic signature that highlighted mTOR signaling as an orchestrator of leukocyte trafficking through the lymphatic barrier of CD patients. Moreover, we demonstrated that LPMC transmigration through the lymphatic endothelium of patients with CD depends on the capability of mTOR to trigger interleukin 20 receptor subunit α (IL20RA)-mediated intracellular signaling. Conclusively, our study suggests that leukocyte trafficking through the intestinal lymphatic microvasculature can be controlled by modulating IL20RA, thus leading to the resolution of chronic inflammation in patients with CD.

## 1. Introduction

Crohn’s disease (CD), belonging to inflammatory bowel disease (IBD), is a chronic inflammatory condition that can affect different portions of the gastrointestinal tract [1]. Clinically, CD manifests with persistent diarrhea, rectal bleeding, urgent need to move bowels, and abdominal pain. From a histological point of view, inflammation of the gut mucosa may affect all segments of the gastrointestinal tract, more commonly the terminal ileum and colon, with an asymmetrical, segmental, and transmural pattern. Moreover, patients may develop complications due to chronic inflammation, such as strictures, fistulas, or abscesses, leading to surgical resection of the complicated disease segment [1]. To date, there is no cure for this disease, and current therapeutic strategies only aim at relieving symptoms, thus preventing complications and hampering the progressive course of the disease. Therefore, understanding the cellular and molecular mechanisms underlying CD etiopathogenesis may be of help for novel therapeutic strategies.

The gut is one of the largest reservoirs of immune cells in the organism, which mostly reside in the lamina propria and the mucosal intraepithelial compartment. Human intestinal lymphatic endothelial cells (HILECs) [2] form an intricate vascular network within the lamina propria, owning the physiological function of clearing inflammatory cells, small molecules, and microbiome antigens from the mucosa to gut-associated lymphoid tissue, in order to ensure efficient responses by circulating T and B lymphocytes [3]. For these reasons, lymphatics have always received great attention in CD pathogenesis because of their role in draining inflammatory infiltrate from the mucosa to lymph nodes, eventually resulting as a non-immune cell compartment in charge of resolving chronic inflammation [4,5,6]. Previous studies reported that dysfunctions in the drainage capacity of the lymphatic system occur in human CD bowel strictures [5]. Moreover, lymphatic vessel density was associated with a higher risk of endoscopic disease recurrence after surgery in CD patients, indicating lymphatics as important players in the pathogenesis of chronic intestinal inflammation [7]. We also previously demonstrated that stimulation of lymphatic functions in experimental models of IBD promotes inflammatory cell mobilization and ameliorates the disease course in mice [2], further supporting the concept that efficient lymphatic drainage is essential for the resolution of intestinal inflammation.

Leukocyte transmigration across the lymphatic barrier is tightly regulated by several mechanisms, ranging from the expression on lymphatic endothelial cells of specific adhesion molecules and tight junctions, to the modulation of the expression of signaling molecules and their relative receptors; the combination of all these actors and factors orchestrates immune cell migration from the mucosal compartment to draining lymph nodes through lymphatics. Although extensive literature describing immune cells trafficking across lymphatic vessels is currently available [8,9,10,11], the intracellular signaling that regulates lymphatic vessel permeability and inflammatory cell clearance in CD has never been investigated.

Here, we show for the first time the transcriptional profiling of HILEC isolated from CD patients, emphasizing specific cellular signaling that regulates leukocyte trafficking across the lymphatic endothelial barrier. Our findings highlight new molecular mechanisms that may be useful in the future to develop novel therapeutic strategies for the control of lymphatic drainage in chronic inflammatory conditions, such as CD.

## 2. Material and Methods

### 2.1. Human Intestinal Lymphatic Endothelial Cell (HILEC) Isolation and Culture and Lentiviral Transduction

HILECs were isolated, as previously described [12], from inflamed ilea of *n* = 5 CD patients undergoing surgery, whose characteristics are listed in Table 1. Healthy HILECs were isolated from healthy resection margins of ilea of *n* = 5 patients undergoing surgery for colorectal cancer. HILECs were maintained in culture with endothelial cell growth medium (EGM-2, Lonza) and split every 3 d. Cells were used between passages 2 and 6. The study was approved by the Humanitas Research Hospital ethics committee, and all subjects provided written informed consent.

Healthy and CD HILECs were transduced with lentiviral particles, produced as previously described [12,13], carrying either *Green Fluorescent Protein GFP* (www.addgene.org), *GFP*-tagged *Arachidonate 12-Lipoxygenase, 12R Type (ALOX12B*, OriGene Technologies, Inc., Rockville, MD, USA), *GFP*-tagged *HtrA Serine Peptidase 4 (HTRA4,* Origene), *GFP*-tagged *Interleukin 20 Receptor Subunit Alpha (IL20RA*, Origene), or *GFP*-tagged *Signal Peptide, CUB Domain And EGF Like Domain Containing 2 (SCUBE2*, Origene Rockville, MD, USA) encoding sequences. A lentiviral vector construct encoding *GFP*-tagged *BMP and activin membrane-bound inhibitor homolog (BAMBI*) was generated by PCR amplification of *BAMBI* cDNA with oligonucleotides carrying *BamHI* and *MluI* restriction sites (Primer Bamh1.forward 5′CCGGGATCCGCCACC ATGGACCGGCACAGCAGC 3′; Primer MluI.reverse 5′ CCG ACGCGT GGGGCCCAGAGGGGCC 3′). The PCR product was digested with BamHI and MluI restriction enzymes and cloned into the GFP-tag Origene vector.

### 2.2. RNA Extraction and Sequencing Analysis

RNA was isolated from human CD and healthy HILEC with the RNeasy Mini Kit^®^ (Qiagen, Hilden, Germany) according to the manufacturer’s instructions and stored at −80 °C. Library preparation and RNA-sequencing were performed at Galseq NGS facility (Milan). Read alignments to the hg38 reference genome and gene counts were performed with Spliced Transcripts Alignment to a Reference (STAR) v2.5 [14]. Differential gene expression was carried out with DESeq2 [15]. Functional enrichment for gene ontology (GO) biological processes was performed with the Gene Set Enrichment Analysis (GSEA) software [16] (OSF submission ID an8w3, https://osf.io/an8w3/).

### 2.3. Data Visualization

Heatmaps were performed with GENE-E (www.broadinstitute.org/cancer/software/GENE-E/) by filtering genes based on *p* ≤ 0.05. Illustrations were performed by exploiting the Servier Medical Art website (https://smart.servier.com).

### 2.4. Lamina Propria Mononuclear Cell (LPMC) Isolation

Human lamina propria mononuclear cells (LPMCs) were isolated from the same inflamed surgical ilea of CD patients (*n* = 5) selected to isolate HILECs. Briefly, intestinal mucosa was rinsed three times in HBSS 1X−/− (Euroclone) supplemented with 5% fetal calf serum (FCS). The specimens were then incubated twice in calcium-magnesium-free HBSS + FCS with 1 mM Ethylenediaminetetraacetic acid (EDTA) for 30 min at 37 °C on rotation to discard intestinal epithelial cells. Mucosal specimens were washed with HBSS + 5 mM CaCl_2_ in order to ensure the complete removal of EDTA. Then, intestinal mucosa was incubated in an enzymatic solution composed of RPMI medium with 0.75 mg/mL collagenase type 2 (Worthington Industries, Columbus, OH, USA), 20 μg/mL DNAse (Roche, Basel, Switzerland), supplemented with FCS 5% at 37 °C for 20–30 min to guarantee complete digestion. Then, tissues were filtered with 100 μm and then with 70 μm cell strainers. Cells were washed twice with RPMI + 5% FCS and underwent Ficoll (CEDERLANE, Burlington, ON, Canada) density gradient centrifugation for 30 min at 400 g without brake. The LPMC-containing ring (interphase) was carefully removed and washed with RPMI. Cells were then counted and freshly used for transmigration experiments.

### 2.5. Transwell-Based Transmigration Assay

Either CD or healthy HILECs, unmodified or genetically modified to overexpress either ALOX12, BAMBI, HTRA4, IL20RA, SCUBE2, or GFP (control), were seeded on a fibronectin-coated 6-well transwell permeable membranes with 3 μm pore size (Corning Costar, Cambridge, MA, USA) and cultured in endothelial cell growth medium (EGM-2, Lonza, Basel, Switzerland). HILEC monolayers were then treated with 100 nM rapamycin (Sigma-Aldrich, St. Louis, MI, USA) or left untreated. Upon 17 h incubation, rapamycin was washed out, and 5 × 10^5^ LMPCs from CD patients (*n* = 5) were seeded on HILEC monolayers for 24 h. LPMCs that had transmigrated in the lower chamber of the transwell were then collected and counted.

### 2.6. Statistical Analysis and Data Visualization

Statistical analysis was performed with Microsoft Excel, IBM SPSS and Graph Pad 7 Prism statistical packages. Data were presented as means ± s.e.m. Mean comparisons were analyzed with two-tailed unpaired Student’s t-test or a two-way ANOVA with a two-stage linear step-up procedure of Benjamini, Krieger, and Yekutieli for multiple comparisons. *P*-values lower than or equal to 0.05 were considered statistically significant. Genes shared among the enriched pathways were obtained by Venn diagrams created using JVenn software (http://jvenn.toulouse.inra.fr/).

## 3. Results

### 3.1. Crohn’s Disease (CD) HILECs Display Peculiar Transcriptomic Signatures

To define the molecular signatures associated with CD lymphatic endothelial cells, HILECs were isolated either from inflamed ilea of patients with CD who underwent surgery (CD HILEC), or from healthy resection margins of ilea collected from patients with colorectal cancer (healthy HILEC) (Table 1).

**Figure 1:**
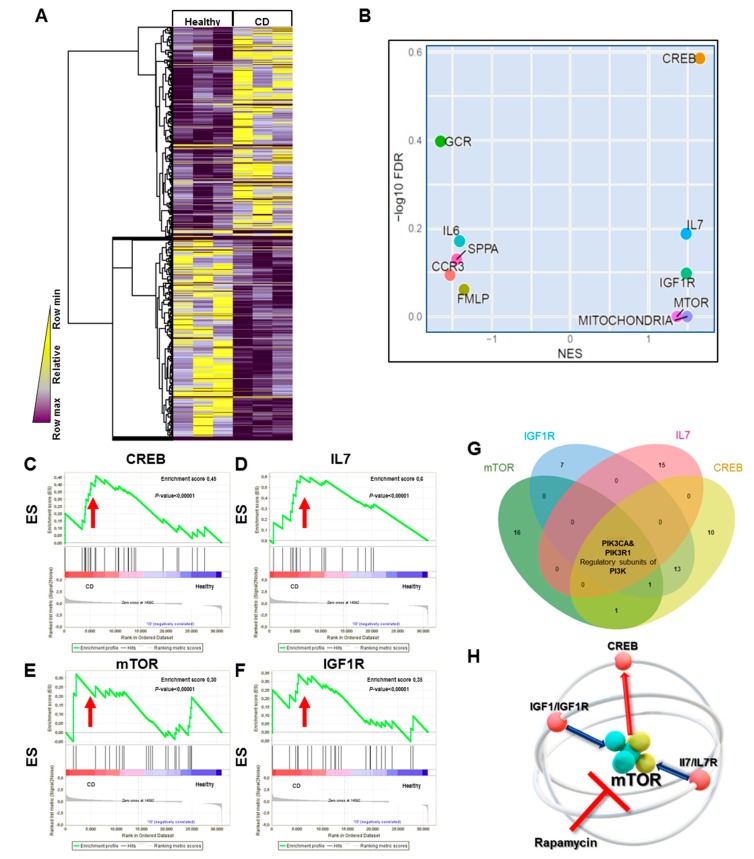
CD HILECs display peculiar transcriptomic signatures. (**A**) Heatmap showing differentially modulated genes with statistical significance (*p* ≤ 0.05) in CD HILEC versus healthy cells (*n* = 3 patients/group). (**B**) Scatter plot visualizing gene set enrichment analysis (GSEA) of transcriptomic results according to statistical significance (log10 false discovery rate (FDR), y-axis) as a function of the normalized enrichment scores (NES, x-axis). C–F. GSEA enrichment plots of gene sets in CD HILECs compared to healthy cells, showing enrichment scores (ESs) for CREB (**C**), IL7 (**D**), mTOR (**E**), and IGF1R (**F**). (**G**) Venn diagram showing the intersection among genes belonging to the indicated pathways. (**H**). Cartoon showing the interaction among the indicated pathways.

Transcriptomic analysis by RNA sequencing highlighted a CD HILEC-specific gene expression profile, which is summarized in Figure 1A. The heatmap shows two clusters of significantly upregulated and downregulated genes in CD versus healthy HILEC (Figure 1A), leading to dysregulation of several important pathways identified by Gene Set Enrichment Analysis (GSEA). Among the downregulated pathways, we found those related to glucocorticoid receptor (*GCR*), interleukin 6 (*IL6*), signaling pathway involved in platelet activation (*SSPA*), C-C chemokine receptor type 3 (*CCR3*), and N-formylmethionyl-leicyl-phenylalanine (*FMLP*) (Figure 1B, Figure S1A–E). Among the upregulated, we found *CREB, IL7, IGF1R*, and *mTOR*-related pathways harbored the highest number of differentially expressed genes (Figure 1B–F).

In order to find a possible target to be modulated, we extrapolated the list of genes belonging to *CREB, IL7, IGF1R* and *mTOR* data sets. Using Venn diagrams, these pathways resulted to be linked with each other, as they shared two genes encoding the regulatory subunits of *PI3K*, *PIK3CA*, and *PIK3R1* (Figure 1G). It has been largely reported that both IGF1/IGF1R and IL7/IL7R signaling contribute to mTOR activation [17,18], and that mTOR is associated with the activation of the CREB pathway via AKT signaling [19]. Therefore, in this scenario, we reasoned that, by targeting mTOR, all the other pathways might be simultaneously affected in HILEC, independently from their inflammatory state (Figure 1H).

### 3.2. mTOR Pathway Oversees LPMC Translocation Across CD HILEC

**Figure 2:**
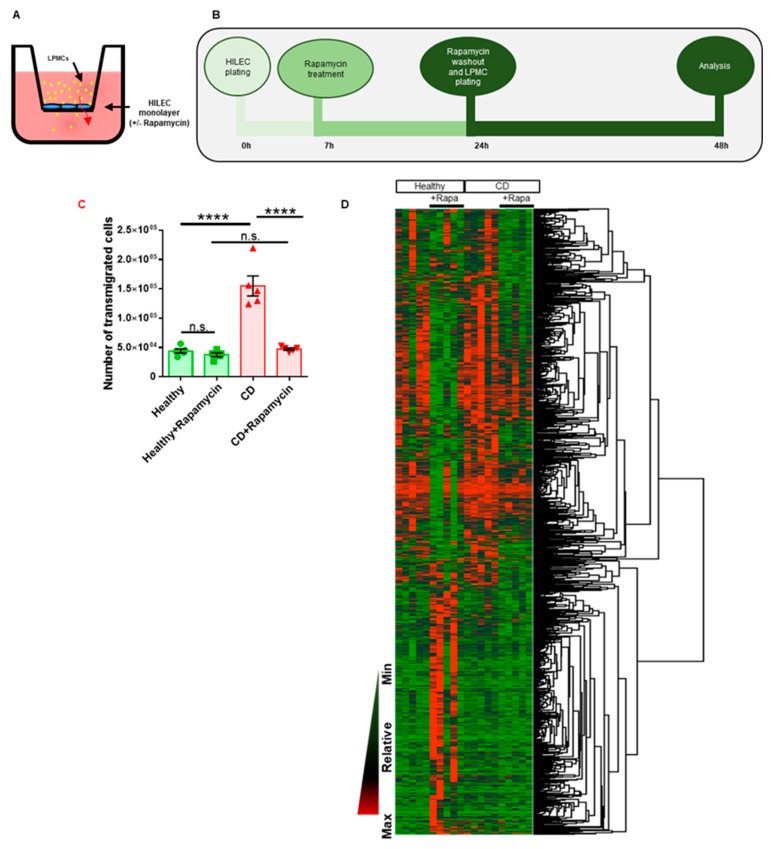
mTOR pathway oversees LPMC transmigration across CD HILECs. (**A**) Graphical representation of the transwell-based assay. (**B**) Schematic description of CD and healthy HILEC treatments with rapamycin during the transmigration assay. (**C**) Scatter plot with bars showing the number of transmigrated LPMCs at the indicated conditions. (**D**) Heatmap showing variations of gene expression in CD and healthy HILECs with or without rapamycin treatment. N = 5/group. Data are represented as mean ± s.e.m. **** *p* ≤ 0.0001, n.s. not significative.

mTOR is largely known to be a critical regulator of immune function, with its inhibitor rapamycin being widely used as an immunosuppressant [20]. In fact, mTOR not only has a direct impact on several biological functions of adaptive immune cells (i.e., T and B cells and antigen-presenting cells) but also orchestrates leukocyte trafficking across the endothelial barrier by controlling the expression of adhesion molecules and chemokines [21]. Recently, Buerger and colleagues observed that, after rapamycin treatment, the number of innate and adaptive immune cells that migrated from the inflamed skin to draining lymph nodes was reduced in a psoriatic mouse model [22]. This suggests not only that mTOR may modulate the migratory capability of immune cells, but that this pathway, according to our RNAseq analysis on CD HILEC, might also affect lymphatic vessel permeability. Starting from these premises we sought to evaluate whether CD HILEC differed from the healthy counterparts in controlling immune cell transmigration, and whether mTOR supervised this function. To pursue this aim, we set up a transwell-based assay in which either CD or healthy HILEC monolayers, plated on the upper chamber of the transwell, were treated with rapamycin or left untreated (Figure 2A,B). Upon 17 h incubation, rapamycin was washed out to exclude any effect on leukocytes, and lamina propria mononuclear cells (LPMCs) isolated from the same CD patient were seeded on HILEC monolayers and left to transmigrate for 24 h in the absence of the compound. Notably, to exclude any variability associated with the inflammatory state of leukocytes from CD patients versus those from healthy controls, we decided to use LPMCs from CD patients also on healthy HILEC. Results showed that the number of LPMCs transmigrated through untreated CD HILEC was higher than those that passed across untreated healthy HILEC (Figure 2C). Interestingly, rapamycin treatment completely reverted this result in CD HILEC and not in healthy cells (Figure 2C), demonstrating that CD HILECs are more permeable to LPMCs than the healthy counterparts, and that the higher number of transmigrated LPMCs through CD HILEC depend on the mTOR pathway.

### 3.3. mTOR-Dependent IL20RA Expression Regulates LPMC Transmigration Across CD HILECs

Since inhibition of the mTOR pathway has been shown to robustly influence LPMC translocation across CD lymphatic monolayers, we sought to further investigate this biological aspect at the transcriptomic level. CD and healthy HILECs, treated or not with rapamycin, were collected and processed for RNA-Seq profiling after 17 h incubation with the compound. GSEA confirmed mTOR downregulation either in rapamycin-treated healthy HILECs (*p*-value not significant, Supplementary Figure S2A) and in rapamycin-treated CD HILECs (*p* = 0.04, Supplementary Figure S2B). Moreover, hierarchical clustering showed a statistically significant modulation of gene expression between healthy and CD HILECs, untreated or not with rapamycin (Figure 2D), indicating a relevant transcriptional variation due to mTOR inhibition. Notably, mTOR inhibition promoted the downregulation of *CREB*, *IL7*, *IGF1,* and *AKT* signaling in both healthy and CD HILECs (Supplementary Figure S3A,B), supporting our hypothesis that mTOR is a molecular hub controlling specific pathways in HILECs (see Figure 1H).

**Figure 3:**
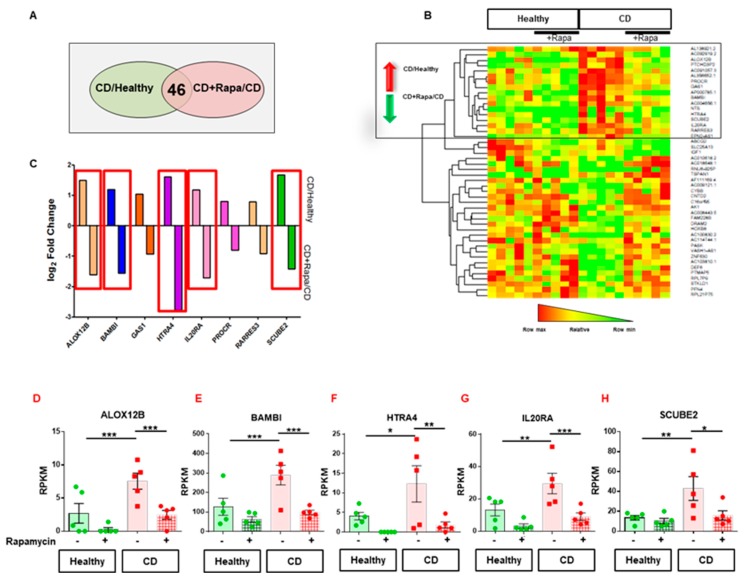
Rapamycin impacts on the CD HILEC transcriptomic state. (**A**) Venn diagram showing genes shared between the indicated groups. (**B**) Heatmap showing the 46 genes concomitantly upregulated in CD versus healthy HILECs and downregulated by rapamycin. (**C**) Bar graphs showing the fold change (log_2_ FC) of the indicated genes in CD versus healthy HILECs and in CD + rapamycin versus CD HILECs. D–H. Scatter plots with bars showing Reads Per Kilobase Million (RPKM) variations at the indicated conditions of ALOX12B (**D**), BAMBI (**E**), HTRA4 (**F**), IL20RA (**G**), and SCUBE2 (**H**). N = 5/group. Data are represented as mean ± s.e.m. * ≤0.05, ** ≤0.01, *** *p* ≤ 0.005.

In order to identify CD HILEC-specific molecular targets that could be affected by mTOR inhibition, we focused our attention on genes whose expressions were deregulated in CD versus healthy HILECs and modulated upon rapamycin treatment. Among these, the expression of 385 genes was significantly affected in CD by comparison to healthy cells. To identify the gene set whose expression was dysregulated in CD versus healthy HILECs and concomitantly affected by rapamycin treatment, we integrated, by Venn diagram, the genes differentially expressed in CD HILECs versus healthy cells and those in the CD + Rapa HILEC versus CD lymphatic endothelial cells (Figure 3A). Results showed 46 genes were shared by the two groups and were likely in charge of regulating CD HILEC-specific functions in an mTOR-dependent manner (Figure 3A,B).

By hierarchical clustering of these 46 genes, we identified a group of genes upregulated in CD versus healthy HILEC that decreased after rapamycin treatment, which might be targeted to modulate lymphatic functions, such as leukocyte transmigration (Figure 3B). Among them, we selected a set of protein-encoding transcripts (Figure 3C) showing Reads Per Kilobase Million RPKM ≥ 1, a log_2_ fold change (FC) ≥ 1 for CD versus healthy HILEC, and a concomitant log_2_ FC ≤ −1 for rapamycin-treated CD HILECs versus CD HILECs. Transcripts displaying these features were *ALOX12B, BAMBI, HTRA4, IL20RA*, and *SCUBE2* (Figure 3D–H). Of note, the expression of these genes was not affected by rapamycin treatment in healthy HILECs, pointing out them as targets specific for CD cells.

**Figure 4:**
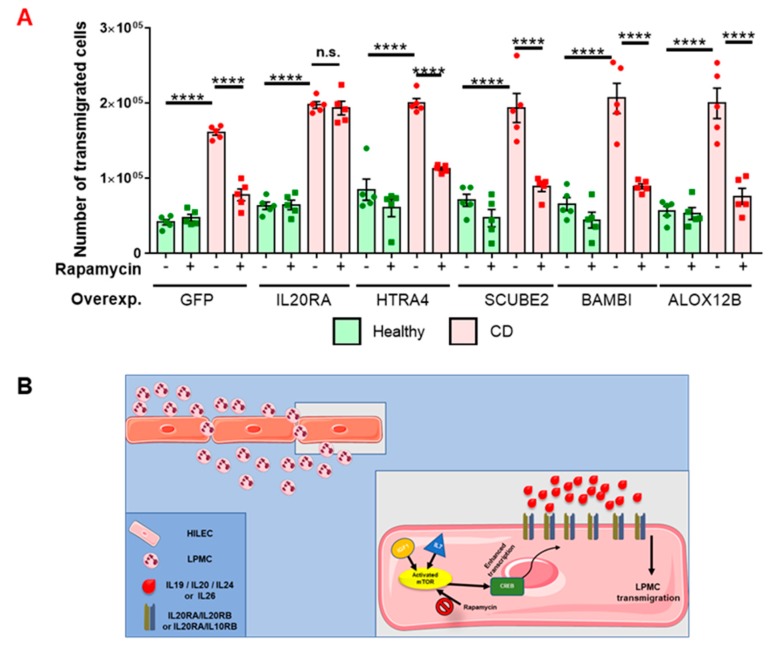
mTOR-dependent IL20RA signaling regulates LPMC transmigration across CD HILECs. (**A**) Scatter plots with bars showing the number of transmigrated LPMCs at the indicated conditions. N = 5/group. Data are represented as mean ± s.e.m. **** *p* ≤ 0.0001, n.s.: not significant. (**B**) Cartoon showing the working model overseeing LPMC transmigration across the lymphatic barrier in patients with CD.

To validate these genes as key players for the regulation of LPMC transmigration across CD HILECs, we performed transwell-based assays with CD LPMCs allowed to transmigrate across both healthy and CD HILECs, genetically modified to overexpress either *ALOX12B, BAMBI, HTRA4, IL20RA, SUCBE2*, or *GFP* (control), in the presence or absence of rapamycin. As expected, control GFP-transduced CD HILECs were more prone to be crossed by LPMCs in comparison to healthy HILECs; this phenotype was completely reverted upon mTOR pathway inhibition (Figure 4A). Most importantly, while the over-expression of *HTRA4, SCUBE2, BAMBI*, and *ALOX12B* did not abolish the effect of rapamycin treatment on CD HILECs, *IL20RA* over-expression blocked the mTOR-dependent increase of LPMC transmigration through CD HILECs versus healthy cells. Notably, rapamycin treatment did not have any effect on LPMC transmigration across healthy HILECs, confirming our previous finding (see Figure 2C). These data demonstrated that LPMC mobilization through the lymphatic endothelium depends on mTOR, via *IL20RA* signaling, ultimately pointing to this receptor as a potential therapeutic target for the modulation of lymphatic drainage in the gut of patients with CD (Figure 4B).

## 4. Discussion

The study here proposed showed, for the first time, that mTOR signaling, known to play important roles in both innate and adaptive immune responses [20], acts not only directly on immune cells in IBD, as previously described [23], but also indirectly through the intestinal lymphatic barrier. In fact, we found the mTOR pathway to be responsible for the leukocyte trafficking across the gut lymphatic endothelium isolated from CD patients, in an *IL20RA*-dependent manner.

The contribution of mTOR to CD pathogenesis is not new; in fact, recent works have predicted mTOR signaling as a driver of intestinal inflammation [24]. While mTOR plays various roles that are related to IBD pathogenesis, including control of immune differentiation and autophagy [25,26], recently Lyons and colleagues found the mTOR pathway also regulated the differentiation state of intestinal epithelial cells, sustaining chronic inflammation [24]. Our findings, involving mTOR signaling also in the regulation of intestinal lymphatic functions, are a new concept that has never been explored, and that point out this pathway as a good therapeutic target to treat CD. Nevertheless, mTOR inhibitors have shown limited efficacy in unselected clinical trials for adult IBD patients [27], although rapamycin has shown clinical efficacy in individual adult cases and in a study of pediatric IBD patients [28,29]. A possible explanation for this failure may be due to the fact that mTOR orchestrates multiple signaling pathways, as also observed in our RNA sequencing data, and there may be collateral mechanisms that override or compensate the inhibitory effects of rapamycin, which eventually may result in being ineffective. However, this is only speculation, and future investigations aimed at the identification of mTOR-regulated pathways are urgently needed to develop drugs that, by directly or indirectly controlling mTOR, may have better outcomes in terms of therapeutic efficacy in patients with CD. IL20RA, which we found to mediate the mTOR-dependent leukocyte transmigration through CD HILECs, could be a good candidate in this sense. The *IL20RA* gene encodes for the subunit α of the heterodimeric receptor IL20RI, consisting of this subunit and IL20 subunit β. This type of receptor transduces IL-19, IL20, IL-24, and IL-26 cytokine signaling, eventually activating Janus kinase-signal and transcription pathways [30]. Interestingly, *IL20RA* was found to be upregulated in other immune-mediated diseases such as psoriasis and rheumatoid arthritis [22,23,24], pointing out these subunits as important for inflammatory diseases. Therefore, it is reasonable that the IL20R axis may intervene also in CD pathogenesis.

A decade ago, a study performed by Hammer and colleagues demonstrated that IL-20 in lymphatic endothelial cells promoted their proliferation, tubule formation, and migration capabilities, which are hallmarks of lymphangiogenic properties in vitro. Moreover, both IL20-induced cell migration and capillary formation were dependent on mTOR activity, thus demonstrating the pivotal role of the mTOR–Akt signaling axis in lymphatic endothelial cells [31,32,33]. This previous evidence further supports our novel findings that highlight IL20RA as a mediator of lymphatic endothelial cell functions, such as leukocyte trafficking via the mTOR pathway. However, additional studies are required to further investigate the molecular mechanism behind this process.

In summary, the mTOR pathway, together with *IGF1*- and *IL7*-mediated signaling, was activated in CD HILECs, eventually promoting *IL20RA* transcription likely via activation of the CREB pathway. *IL20RA*, known to heterodimerize with either *IL20RB* or *IL10RB*, senses IL20 (or IL19, IL24, or IL26) cytokines, which, in turn, initiates a cascade signaling that promotes LPMC transmigration across the intestinal lymphatic barrier (Figure 4B).

**Figure 5:**
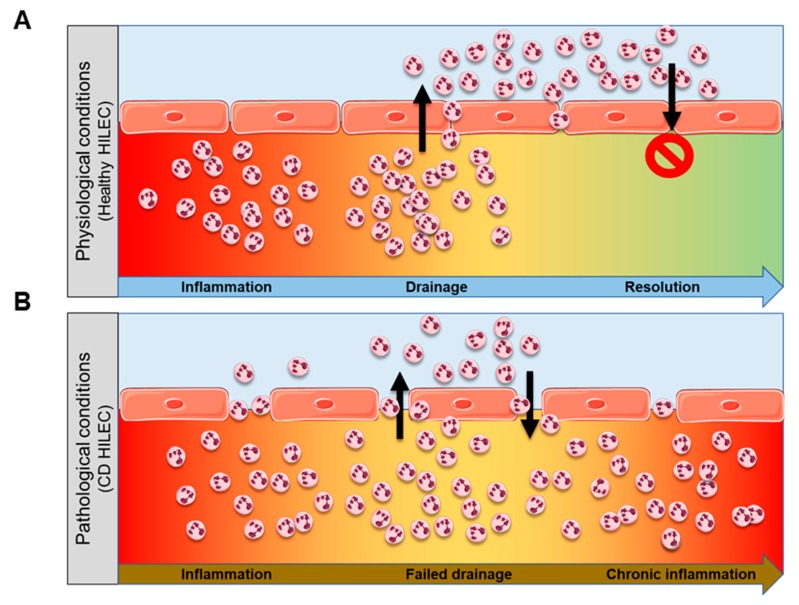
Lymphatic barrier is compromised in CD. Cartoons showing lymphatic drainage in physiological conditions (**A**) and in CD (**B**).

In physiological conditions, during the inflammatory response, lymphatics have the role to drain immune cells from inflamed mucosa to lymph nodes, leading to clearance of leukocyte infiltrates and to the resolution of inflammation (Figure 5A). On the contrary, in patients with CD, lymphatic drainage is compromised [5] due to high levels of mTOR and mTOR-dependent signaling pathways, leading to the accumulation of immune infiltrate within intestinal mucosa, which fosters chronic inflammation (Figure 5B). Our study sheds light on additional molecular mechanisms behind the process that promotes a “leaky” lymphatic barrier in patients with CD.

From a clinical point of view, looking at mTOR as a potential therapeutic target might be challenging because of its relevance in many cellular processes; in fact, its inhibition might subvert physiological functions of the organism, and this may explain why drugs targeting mTOR have shown inefficacy in treating patients with CD. The identification of molecules, such as IL20RA, that are directly or indirectly modulated by mTOR and that may have more cell-specific and/or tissue-specific functions, could be more effective in restoring a proper lymph drainage and an appropriate mobilization of inflammatory cells from the active mucosa to the draining lymph nodes, ultimately leading to the resolution of chronic inflammation in patients with CD.

## Figures and Tables

**Table 1:**
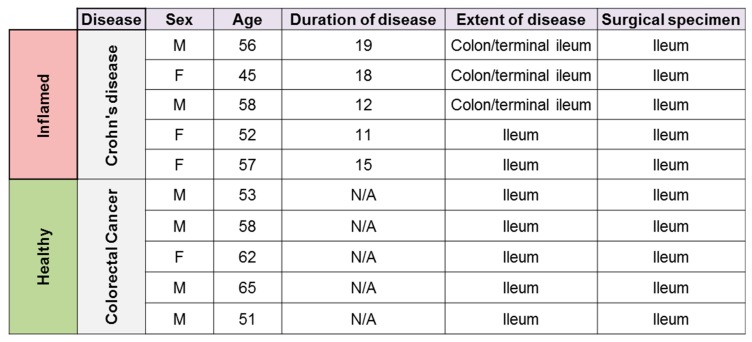
Characteristics of enrolled patients with active Crohn’s Disease (CD) and patients with colorectal cancer, whose healthy ilea were used to derive control human intestinal lymphatic endothelial cells (HILECs).

## Supplementary Materials

The following are available online at https://www.mdpi.com/2073-4409/8/8/924/s1. Supplementary Figure S1. Differential gene expression analysis of CD HILECs. Supplementary Figure S2. Differential gene expression analysis of CD and healthy HILECs upon rapamycin treatment. Supplementary Figure S3. Differential gene expression analysis of CD HILECs after rapamycin treatment.
