# Supplementary material for: mTOR-Dependent Stimulation of IL20RA Orchestrates Immune Cell Trafficking through Lymphatic Endothelium in Patients with Crohn’s Disease
Source: Cells. 2019 Aug 18;8(8):924. doi: 10.3390/cells8080924 (PMC6721646; doi:10.3390/cells8080924)
Supplement: Supplementary file 1 [file cells-08-00924-s001.zip › Supplementary Figure 3.pdf]

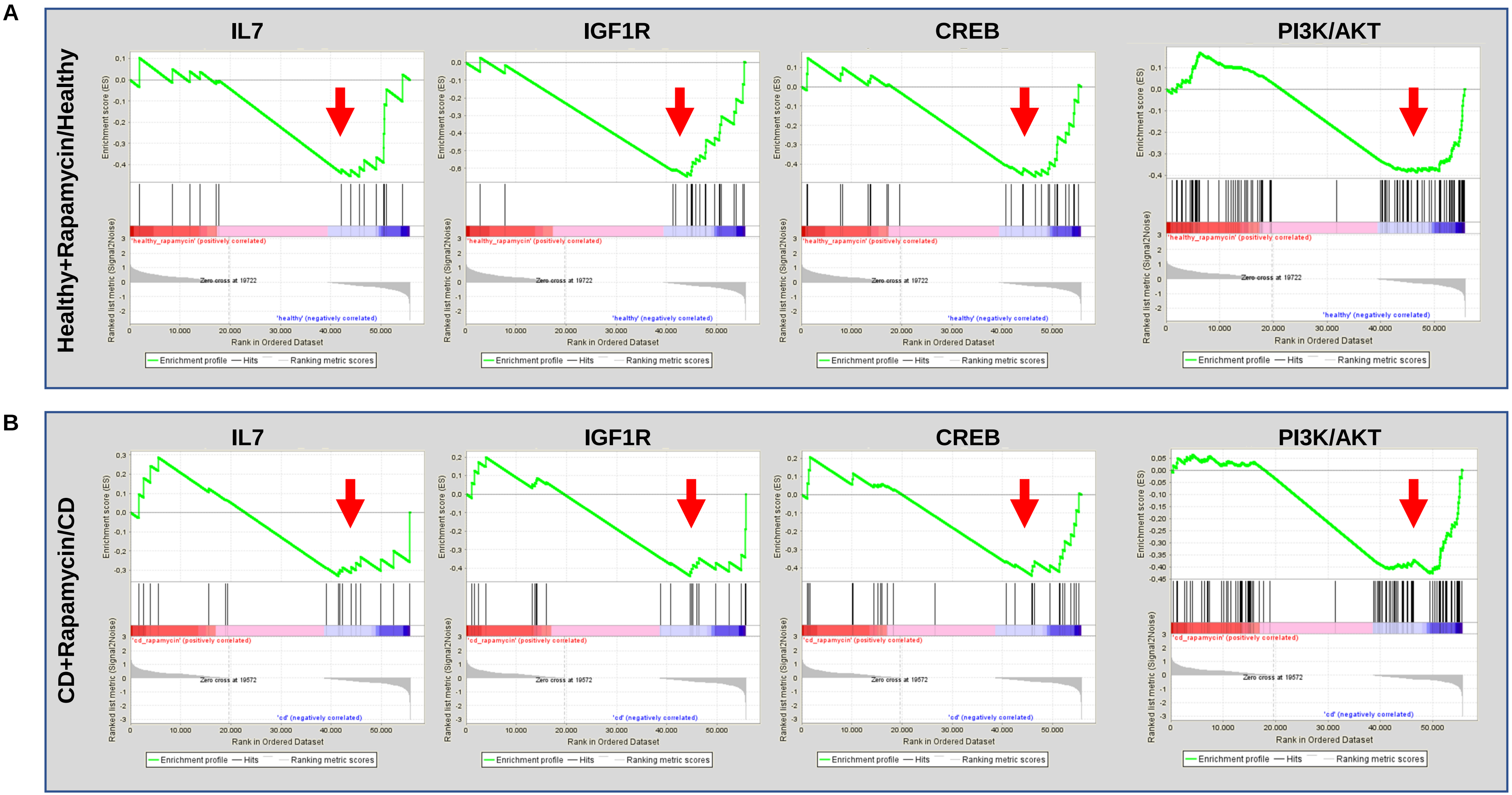

Supplementary Figure 3. Differential gene expression analysis of CD HILEC after Rapamycin treatment. A,B.GSEA enrichment plots of gene sets in CD HILEC by comparison with healthy cell (A) and of CD HILEC+Rapamycin treatment versus CD HILEC (B), (A), CCR3 (B), SPPA (C), FMLP (D) and IL6 (E). The arrows indicate the downregulation of gene sets.
