# Supplementary material for: mTOR-Dependent Stimulation of IL20RA Orchestrates Immune Cell Trafficking through Lymphatic Endothelium in Patients with Crohn’s Disease
Source: Cells. 2019 Aug 18;8(8):924. doi: 10.3390/cells8080924 (PMC6721646; doi:10.3390/cells8080924)
Supplement: Supplementary file 1 [file cells-08-00924-s001.zip › Supplementary Figure 2.pdf]

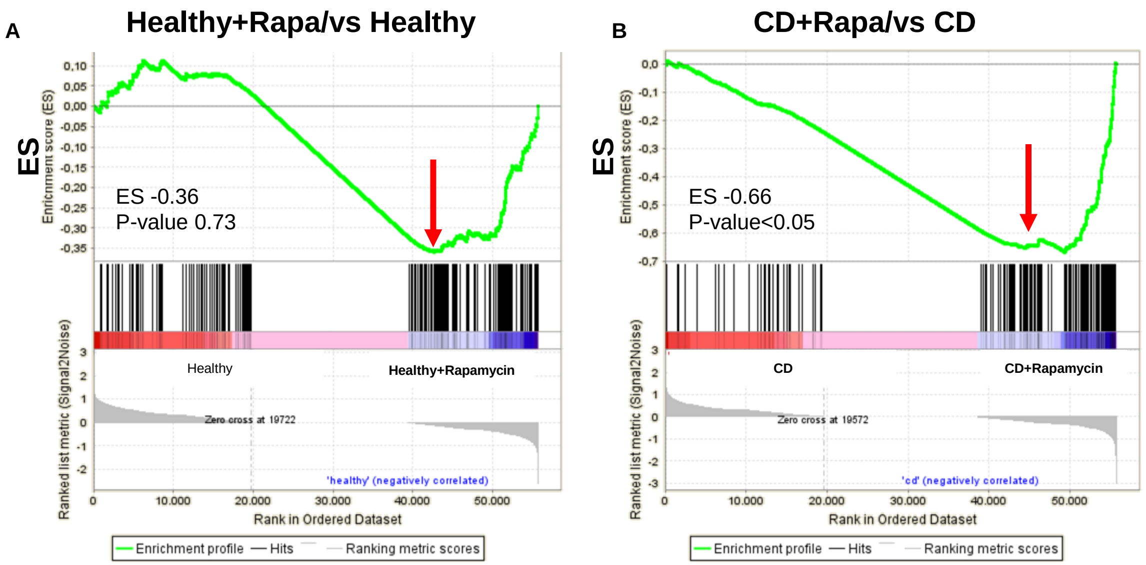

Supplementary Figure 2. Differential gene expression analysis of CD and healthy HILEC upon Rapamycin treatment. A, B.GSEA enrichment plots showing mTOR pathway down-regulation in Healthy+Rapamycin versus Healthy HILEC (A) and in CD+Rapamycin versus CD HILEC (B). The arrows indicate the down-regulation of gene sets.
